# Assessment of real life eating difficulties in Parkinson’s disease patients by measuring plate to mouth movement elongation with inertial sensors

**DOI:** 10.1038/s41598-020-80394-y

**Published:** 2021-01-15

**Authors:** Konstantinos Kyritsis, Petter Fagerberg, Ioannis Ioakimidis, K. Ray Chaudhuri, Heinz Reichmann, Lisa Klingelhoefer, Anastasios Delopoulos

**Affiliations:** 1 Multimedia Understanding Group, Information Processing Laboratory, Department of Electrical and Computer Engineering, Aristotle University of Thessaloniki, Thessaloniki, Greece; 2 Innovative Use of Mobile Phones to Promote Physical Activity and Nutrition Across the Lifespan (the IMPACT) Research Group, Department of Biosciences and Nutrition, Karolinska Institutet, Stockholm, Sweden; 3 King’s College Hospital and King’s College, London, UK; 4 Department of Neurology, Technical University Dresden, Dresden, Germany

**Keywords:** Computational science, Parkinson's disease

## Abstract

Parkinson’s disease (PD) is a neurodegenerative disorder with both motor and non-motor symptoms. Despite the progressive nature of PD, early diagnosis, tracking the disease’s natural history and measuring the drug response are factors that play a major role in determining the quality of life of the affected individual. Apart from the common motor symptoms, i.e., tremor at rest, rigidity and bradykinesia, studies suggest that PD is associated with disturbances in eating behavior and energy intake. Specifically, PD is associated with drug-induced impulsive eating disorders such as binge eating, appetite-related non-motor issues such as weight loss and/or gain as well as dysphagia—factors that correlate with difficulties in completing day-to-day eating-related tasks. In this work we introduce Plate-to-Mouth (PtM), an indicator that relates with the time spent for the hand operating the utensil to transfer a quantity of food from the plate into the mouth during the course of a meal. We propose a two-step approach towards the objective calculation of PtM. Initially, we use the 3D acceleration and orientation velocity signals from an off-the-shelf smartwatch to detect the bite moments and upwards wrist micromovements that occur during a meal session. Afterwards, we process the upwards hand micromovements that appear prior to every detected bite during the meal in order to estimate the bite’s PtM duration. Finally, we use a density-based scheme to estimate the PtM durations distribution and form the in-meal eating behavior profile of the subject. In the results section, we provide validation for every step of the process independently, as well as showcase our findings using a total of three datasets, one collected in a controlled clinical setting using standardized meals (with a total of 28 meal sessions from 7 Healthy Controls (HC) and 21 PD patients) and two collected in-the-wild under free living conditions (37 meals from 4 HC/10 PD patients and 629 meals from 3 HC/3 PD patients, respectively). Experimental results reveal an Area Under the Curve (AUC) of 0.748 for the clinical dataset and 0.775/1.000 for the in-the-wild datasets towards the classification of in-meal eating behavior profiles to the PD or HC group. This is the first work that attempts to use wearable Inertial Measurement Unit (IMU) sensor data, collected both in clinical and in-the-wild settings, towards the extraction of an objective eating behavior indicator for PD.

## Introduction

Parkinson’s disease (PD) is a complex neurological disorder, associated with a number of motor and non-motor symptoms (NMS) such as tremor, bradykinesia, rigidity, problems with manual dexterity, micrographia, cognitive issues, sleep abnormalities and depression^1^. Regardless of PD being incurable, early diagnosis can have a huge impact on the progression of the disease, the patient’s quality of life^2,3^ as well as from a socioeconomic standpoint^4,5^. At the same time, PD diagnosis suffers from high misdiagnosis rates^6,7^. In spite of improved scales and diagnostic criteria for PD^8^, the process is largely subjective and an objective diagnostic tool remains an unmet need.

Over the years, a number of works have associated PD with variations in eating behavior^9–11^. Such variations encompass body weight alterations^10,12,13^, binge eating^11,14,15^ and malnutrition^16^, indicators that are often overlooked^17^. It should be noted that despite the large interest shown towards the eating behavior dimension of PD, only a limited amount of studies attempt to measure such effects objectively. In a recent study, researchers attribute the alteration of eating patterns to levodopa^18^, one of the main medications provided to PD patients. Whichever the cause of eating behavior variations may be, the comorbidity of weight loss and malnutrition can lead to increased frailty^19^, reduced immune function^20^ and synergize against the life quality of the PD patient^16,21^.

The Unified Parkinson’s Disease Rating Scale (UPDRS) is the most widely used and tested scale^22^. The scale consists of four components. Parts I and II deal with non-motor and motor experiences of daily life respectively, Part III is the motor examination and Part IV concerns with motor complications. It should be emphasized that Part II is designed to be a *self-administered questionnaire* and as a result it does not require input from the investigator^23^. Out of all the items in the UPDRS, only two are directly related to eating activities, one is swallowing (i.e., dysphagia) and the other is cutting food and handling utensils, with both of them belonging to Part II. Consequently, recent studies show that dysphagia can be under-diagnosed due to lack of objective tools and/or poor self-awareness^24^.

In this paper, we investigate how the eating behavior and the microstructure of meals can be used as an objective indicator for PD. This is achieved by measuring the upwards hand movements that occur before each food intake moment (i.e., bite) during the course of a meal. Towards this, we introduce Plate-to-Mouth (PtM). More specifically, the PtM indicator is defined as the time spent for the hand operating the utensil (fork or spoon) to transfer a *ready-to-be-consumed* quantity of food from the plate upwards into the mouth. However, in order for such spoonful to be considered as ready-to-be-consumed, two preconditions must be fulfilled. Initially, the utensil has to be loaded. In addition, the eater does not further interact and/or alter the weight or the shape of the spoonful in a voluntary fashion. Food deductions caused by misplacing food on top of the utensil or due to other involuntary factors are allowed. Contribution to the bite’s PtM begins by moving the hand operating the utensil *upwards* with the intention of placing food into the mouth. Upwards hand movements that do not aim at placing food into the mouth, such as upwards movements that may occur during social meals, do not contribute towards the calculation of the bite’s PtM.

In order to approximate PtM, we propose a two stage method that makes use of the 3D acceleration and orientation velocity signals of a typical smartwatch. The first part of the method deals with the recognition of wrist micromovements that occur during the course of a meal (pick food, upwards, mouth, downwards, no movement) using a Support Vector Machine (SVM) array. Additionally, by modeling the temporal evolution of the recognized wrist micromovements the meal’s bite moments can be detected by means of a Recurrent Neural Network (RNN) with two Long-Short Term Memory (LSTM) cells. In the second part of the algorithm the detected upwards micromovements and bite moments are processed using signal processing techniques and the final PtM periods are obtained. Experimental results are presented using three different datasets, one collected in the clinic under controlled conditions (EBePa-at-Clinic) and two collected in-the-wild under free-living conditions (EBePa-at-Home and SData-at-Home), that contain meal sessions from PD patients and healthy controls (HC).

The path leading to the work presented herein was paved by two recent studies of our group^25,26^. Both studies investigated eating behavior variations in the PD population using data collected in a controlled clinical setting; one using manual video annotations and food weight measurements^25^ to objectively measure differences in energy intake between HC, early and advanced-stage PD patients, and one using smartwatch sensor data^26^ presenting a less refined version of the PtM indicator. To this day, this is the first study that attempts to use wearable Inertial Measurement Unit (IMU) sensor data collected both under controlled conditions and in-the-wild towards the extraction of an objective eating behavior-related indicator for PD.

The rest of the paper is organized as follows. In "Related work" section we present a review of the relevant literature regarding the objective measurement of PD symptoms using sensors. An in-depth presentation of the proposed PtM extraction algorithm is provided in "Methods" section. Next, the "Evaluation" section presents the performed experiments. A detailed description of the used datasets is presented in the "Datasets" section. Sections "Results" and "Discussion", present and discuss the obtained results, respectively. Finally, the paper concludes with "Conclusions" section.

## Related work

A great amount of studies exist in the literature that deal with the detection of PD motor and non-motor symptoms in an objective fashion under clinical or controlled settings. Such works use a variety of sensors (e.g., cameras, IMU and microphones) to investigate tremor manifestations^27,28^, dyskinesia^29^, speech impairment^30,31^, gait^32^ and sleep disorders^33–36^.

The recent boom in the commercial portable and wearable devices enabled the research community to develop unobtrusive solutions for PD capable of obtaining objective measurements in-the-wild (i.e., outside of controlled clinical environments)^37,38^. For example, the works of Papadopoulos *et al.*^39,40^ showcase a method towards the detection of tremorous episodes using the 3D acceleration information captured from a typical smartphone during voice calls. The authors mention the label uncertainty problem and suggest a deep Multiple Instance Learning Convolutional Neural Network (MIL-CNN) as a solution. The problem of label uncertainty in-the-wild has also been raised in another work^41^ that deals with the detection of dyskinesia using off-the-shelf smartwatches. The recent studies presented by Iakovakis *et al.*^42,43^ also use typical smartphones as the sensing platform. More specifically, the authors analyze the patterns that emerge from the finger interaction with the touchscreen during natural typing to detect decline of fine motor skills. Besides using information gathered in-the-wild, one additional constant among the studies mentioned above is that they first extract indicators that describe the targeted PD symptom (e.g., tremor) and then use Machine Learning (ML) approaches in order to classify subjects to the PD or HC populations.

Despite the large number of studies that deal with the detection of PD motor symptoms, only a limited amount of works investigate the alterations of eating behavior in PD patients. In an early study published in 1989, Athlin *et al.*^44^ analyzed video recordings in order to examine the deviant eating behavior in elderly PD patients between the ages of 62 and 83. Their study of 24 subjects (10 demented and 14 non-demented) revealed problems in the eating tasks of: (1) handling food on the plate, (2) transporting food into the mouth, (3) manipulating food into the mouth and (4) swallowing. In more detail, regarding the transportation of food from the plate into the mouth, the authors observed many instances where the patients spilled food from the utensil while mid-air due to tremor. Additionally, cases of “undershooting” (i.e., utensil not reaching the mouth) and patients not inclining their head to meet the approaching utensil were noted. Finally, the hand-arm movement responsible for transferring food from the plate to the mouth was performed stepwise or had to be adjusted. The early work of Athlin may be among the first that initially identified the issue; however, the descriptive analysis and the small sample size do not allow for any concrete conclusions.

In our previous work^25^, we rekindled the topic of experimentally investigating eating behavior alterations in PD patients. We used an extended, video-only, version of the EBePa-at-Home (EaC) dataset that contains a total of 64 subjects, out of whom 23 were HC, 20 were early-stage PD patients (ESPD) and 21 were advanced-stage PD patients (ASPD). It should be noted that the analysis was based solely on manual video annotations (no IMU sensor data) and without any knowledge of wrist micromovements (e.g., upwards). Similar to the EaC data collection protocol, participants initially underwent clinical evaluations and then freely consumed a standardized meal in a controlled setting in front of two cameras. The food was weighted pre-/post-meal in order to calculate EI. The study focused towards the investigation of differences in objectively-measured energy intake (EI) between the HC, ESPD and ASPD populations. In the experimental section, we reported that ASPD have significantly lower energy intake than ESPD and HC, thus indicating an increased risk for weight loss. Additionally, the lower EI among ASPD versus HC could be explained (by $86\%$) due to: (1) higher upper extremity tremor scores, (2) increased subjectively reported eating problems, as well as dysphagia, and (3) performing fewer spoonfuls during the meal. Finally, eating problems, dysphagia and the number of performed spoonfuls could explain approximately half of the observed lower energy intake among advanced versus early-stage PD patients. The work concluded by suggesting that an improvement to the nutritional status of PD patients can lead to an improved quality of life.

## Methods

In this section we will present the process of extracting a meal’s *Plate-to-Mouth* (PtM) periods using the 3D acceleration and orientation velocity signals that originate from a typical smartwatch. The PtM periods extraction is achieved in two steps. In the first step we use an SVM array and an RNN to detect all hand micromovements, model their temporal evolution within the meal and detect the food intake moments (i.e., bites). A number of five micromovement categories are used to model food intakes, more specifically, pick food (“p”), upwards (“u”), mouth (“m”), downwards (“d”) and no movement (“n”). In the context of this work, we will solely focus on the “u” micromovements. In the second step, we process the detected bite moments and “u” micromovements using signal processing techniques with the aim of obtaining the meal’s PtM periods.

### Data pre-processing

Formally, a meal session will be represented by the sensor data matrix $\mathbf{S}=[\mathbf{a}_{x},\mathbf{a}_{y}, \mathbf{a}_{z},\mathbf{g}_{x},\mathbf{g}_{y},\mathbf{g}_{z}]$, with dimensions $N \times 6$. The length of $\mathbf{S}$ is defined as $N = t \, f_s$, where *t* is the total duration of the meal in seconds and $f_s$ the sensor sampling frequency in Hz. The vectors $\mathbf{a}_{x},\mathbf{a}_{y},\mathbf{a}_{z},\mathbf{g}_{x},\mathbf{g}_{y}$ and $\mathbf{g}_{z}$ correspond the triaxial acceleration and gyroscope sensor measurements, respectively. To deal with sensor noise, each sensor stream was smoothed using a 5th order median filter. Moreover, since the acceleration sensor captures the earth’s gravitational field in addition any hand movement, we convolved the acceleration sensor streams ($\mathbf{a}_{x},\mathbf{a}_{y}$ and $\mathbf{a}_{z}$) using a high-pass Finite Impulse Response (FIR) filter with a 512 tap delay line and a cutoff frequency of 1 Hz.

### Upwards micromovement and bite moment detection

In order to detect the bite and upwards micromovement moments we follow the approach published in a previous work of ours^45^. The process begins by using a sliding window of length $w_l=0.2 \, f_{s}$ and step $w_s = 0.1 \, f_s$ samples (i.e., with $50\%$ overlap) to extract frames from the sensor data matrix $\mathbf{S}$. A total of $K=\lfloor (N-w_{l})/w_{s} \rfloor$ frames are extracted, each with dimensions equal to $w_l \times 6$. Subsequently, from each of the $i=1,2,\ldots ,K$ frames we derive *L* temporal and frequency domain features, effectively transforming the $N \times 6$ sensor data matrix $\mathbf{S}$ into the $K \times L$ feature matrix $\mathbf{F}$. In more detail, for each of the sensor streams we calculate the mean, standard deviation, variance, minimum and maximum values, range of values, zero crossing rate, energy and the first $(w_l/2) + 1$ Discrete Fourier Transform (DFT) coefficients. In addition, for each sensor we calculate the simple moving average given by $\frac{1}{w_l} \sum _{i=1}^{w_l} |x(i)| + |y(i)| + |z(i)|$, where *x*(*i*), *y*(*i*) and *z*(*i*) correspond to the *i*th sample of *x*, *y* and *z* streams in a single frame.

Following our previous approach^45^, we address the multiclass, micromovement recognition problem by employing an array of ten binary, one-versus-one SVM classifiers. The number of the one-versus-one classifiers results from $c\; (c-1)/2$, where *c* indicates the total number of classes^46^; in the context of this work $c=5$ (pick food, upwards, mouth, downwards and no-movement), resulting to a total of ten binary classifiers. In essence, the one-vs-one approach splits the multiclass classification problem into one binary problem for each possible pair of classes; e.g., pick food versus upwards, mouth versus downwards, etc. All binary SVM classifiers use the Radial Basis Function (RBF) kernel. By processing each of the *K* feature vectors of matrix $\mathbf{F}$ using the SVM array we obtain the $K \times 10$ score matrix $\mathbf{V}$. Each row of $\mathbf{V}$ contains the ten pairwise prediction scores from the one-versus-one classifiers. Subsequently, each of the ten scores corresponds to the sample’s (i.e., feature vector) distance from the separating hyperplane formed by each of the ten binary SVM classifiers. Furthermore, by applying the max wins voting scheme^46^ to each of the rows of $\mathbf{V}$ we obtain the $K \times 1$ micromovement label vector $\mathbf{y}_{mm}$.

**Figure 1:**
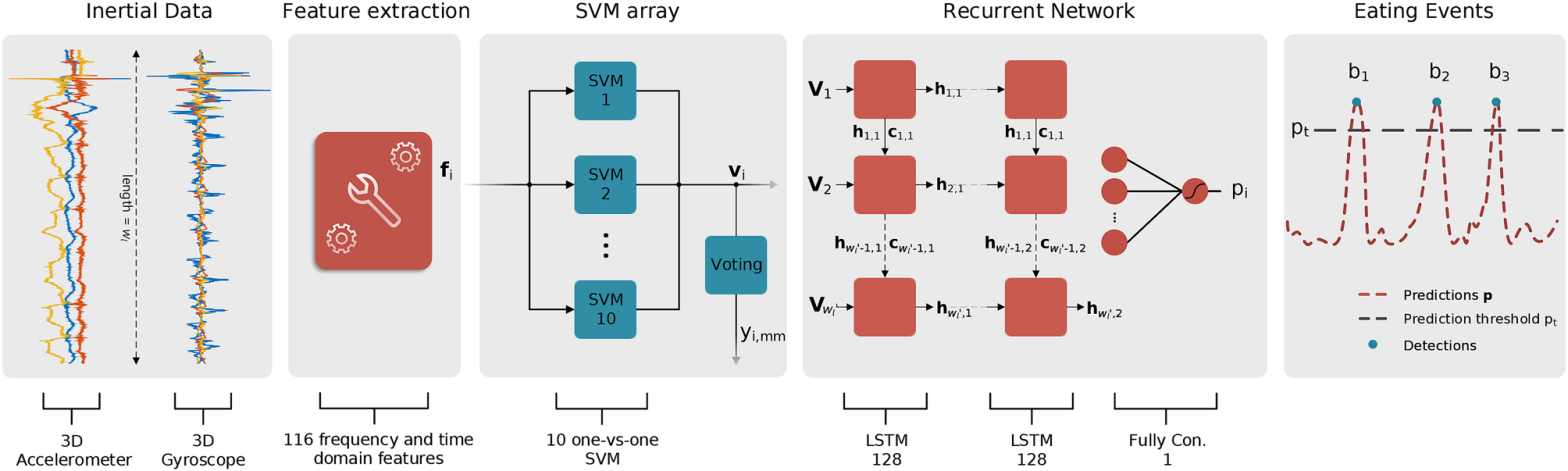
Figure depicting the overall pipeline of the micromovement recognition/bite detection part of the algorithm. From left to right, the windowed 3D accelerometer and gyroscope streams of length $w_{l}$ are transformed into the 116-dimensional feature vector $\mathbf{f}_{i}$. Next, using an array of ten one-versus-one SVM the feature vector $\mathbf{f}_{i}$ is transformed into the 10-dimensional SVM prediction score vector $\mathbf{v}_{i}$. By applying a voting scheme to the one-vs-one scores of the ten SVM (i.e., to the $\mathbf{v}_{i}$ vector) we obtain $y_{i,mm}$ which indicates the micromovement label that corresponds to the *i*th window. Processing of additional sensor windows leads to the creation of the meal’s SVM score matrix $\mathbf{V}$ and label vector $\mathbf{y}_{mm}$. Furthermore, by processing windows of $\mathbf{V}$ with length $w'_{l}$, the RNN outputs the probability ${p}_{i}$ that the given window sequence is a food intake cycle. Variables $\mathbf{h}_{i,j}$ and $\mathbf{c}_{i,j}$ are used to represent the *i*th hidden output and cell state of the *j*th LSTM layer, respectively. The rightmost part of the figure illustrates the local maxima search in the meal’s prediction vector $\mathbf{p}$. Variables $b_{1}$, $b_{2}$ and $b_{3}$ represent three detected bites, while $p_{t}$ represents the prediction threshold.

Bite moment detection is achieved by modeling the temporal evolution of micromovements using an RNN. More specifically, the proposed RNN architecture consists of two Long-Short Term Memory (LSTM) layers, followed by a fully connected layer with a single neuron. Both LSTM layers of the network use 128 hidden cells. The bite moment detection process begins by extracting frames from the micromovement score matrix $\mathbf{V}$ using a sliding window of length $w_{l}'= 3 \, f_s$ and step $w_{s}' = 0.2 \, f_s$ samples. Using this sliding window, a total of $K' = \lfloor (K-w_{l}')/w_{s}'\rfloor + 1$ frames are extracted, each with dimensions $w_{l}' \times 10$. The extracted frames are then propagated to the RNN and the $K'\times 1$ probability vector $\mathbf{p}$ is obtained. The RNN network is trained using as positive samples the micromovement sequences that start with “p”, end with “d” and contain an “m” event and as negative samples all other sequences. Therefore, each of the $i=1,2,\ldots ,K'$ elements of $\mathbf{p}$ indicates the probability that the *i*th input frame is a food intake event. Prior to the bite moment detection, the probability vector $\mathbf{p}$ is thresholded using a threshold $p_{t}$. More specifically, all elements of $\mathbf{p}$ that are less than $p_{t}$ are replaced with zeros. We selected $p_{t}$ to be equal to 0.89 as proposed by a number of previous works from our group^45,47–49^. Finally, the set of detected bite timestamps $\mathfrak{B}=\{b_{1},b_{2},\ldots ,b_{U}\}$, with $U \in \mathbb {Z}^{+}$ indicating the cardinality of $\mathfrak{B}$ (i.e., the number of detected bites), is obtained by performing a local maxima search in $\mathbf{p}$ using a minimum distance of $3\, f_{s}$ samples between consecutive peaks. Figure 1 illustrates the processing pipeline regarding the upwards micromovement and bite moment detection part of the proposed algorithm.

### Plate to Mouth duration extraction

The extraction of a meal’s PtM durations begins by transforming the micromovement label vector $\mathbf{y}_{mm}$ into the binary vector $\mathbf{y}_{u}$. This is achieved by replacing with zeros the elements of $\mathbf{y}_{mm}$ that belong to the “p”, “m”, “d” and “n” classes and with ones the elements that belong to the “u” class. Essentially, $\mathbf{y}_{u}$ is non-zero in the parts of the meal where the wrist wearing the smartwatch performs an upwards movement and zero everywhere else.

The PtM durations of a meal are calculated for all inter-bite intervals, i.e., the periods between two consecutive, detected, bites. The first inter-bite interval is considered as the period between the beginning of the meal and the moment of the first detected bite, i.e., the interval $[0,b_{1}]$. PtM calculation continues by extracting the subset $\mathbf{y}_{i,u}$ of the elements of $\mathbf{y}_{u}$ with timestamps that are within the limits of the *i*th inter-bite interval, denoted by $[b_{i-1},b_{i}]$. Moreover, we will use the term *u-region* to represent a cluster of, one or more, consecutive non-zero elements in $\mathbf{y}_{u}$. The *j*th u-region for the *i*th inter-bite interval can be described by it’s start and end moments $r_{i,j} = [t^{s}_{i,j},t^{e}_{i,j}]$. Given $\mathbf{y}_{i,u}$ we extract the set of u-regions $\mathfrak{R}_{i}=\{[t_{i,1}^{s},t_{i,1}^{e}],\ldots , [t_{i,D-1}^{s},t_{i,D-1}^{e}],[t_{i,D}^{s},t_{i,D}^{e}]\} =\{r_{i,1},\ldots ,r_{i,D-1},r_{i,D}\}$, with $D \in \mathbb {Z}^{+}$ indicating the cardinality of the $\mathfrak{R}_{i}$ set. Since there is no overlap between the u-regions of the set, the elements of $\mathfrak{R}_{i}$ are sorted in an ascending fashion given their starting moments. Starting at the *final* u-region $r_{i,D}$ we search backwards in an attempt to merge it with the previous u-region $r_{i,D-1}$ of the $\mathfrak{R}_{i}$ set. The merge is successful if the distance between the two u-regions, calculated as $d_{i}(r_{i,D},r_{i,D-1}) = t_{i,D}^{s}-t_{i,D-1}^{e}$, is lower than a threshold $\lambda _{d}$. The merging process continues until: (1) we have merged all u-regions in the $\mathfrak{R}_{i}$ set (i.e., there are no more u-regions to merge in the *i*th inter-bite interval), or (2) a merge was not successful (i.e., distance between the two u-regions was greater than $\lambda _{d}$ seconds). For the experiments presented in the "Results" section, we selected $\lambda _{d}$ to be equal to 0.5 s. We support this choice as it approximates the median duration (0.483 s) of non-upwards movements that occur between upwards movements that contribute to the bite’s PtM, according to the video GT of the EaC dataset.

We select as $\tau _{i}^{e}$ the ending moment of the last u-region in the $\mathfrak{R}_{i}$ set (i.e., $t_{i,D}^{e}$). Subsequently, given the number of consecutive successful merges $J_{i}$, we set as $\tau _{i}^{s}$ the starting moment of the last-merged u-region, $t_{i,D-J}^{s}$. In essence, the moment $\tau _{i}^{s}$ corresponds to the timestamp at the *beginning* of the upwards motion, when the wrist operating the utensil begins transferring food towards the mouth area to perform bite $b_{i}$. Additionally, $\tau _{i}^{e}$ corresponds to the timestamp at the *end* of the upwards wrist motion, prior to placing food into the mouth. Finally, the PtM period for the *i*th inter-bite interval $[b_{i-1},b_{i}]$ can be calculated using the formula below.

1a $$\begin{aligned}&\text {PtM}_{i} = \tau _{i}^{e} -\tau _{i}^{s} = t_{i,D}^{e} - t_{i,D}^{s},&\text {if} \quad J_{i}=0 \end{aligned}$$

1b $$\begin{aligned}&\text {PtM}_{i} = \tau _{i}^{e} -\tau _{i}^{s} - \sum _{j=1}^{J}d_{i}(r_{D-j+1},r_{D-j}) = t_{i,D}^{e} - t_{i,D-J}^{s} - \sum _{j=1}^{J}(t_{i,D-j+1}^{s}-t_{i,D-j}^{e}),&\text {if} \quad J_{i}>0 \end{aligned}$$

**Figure 2:**
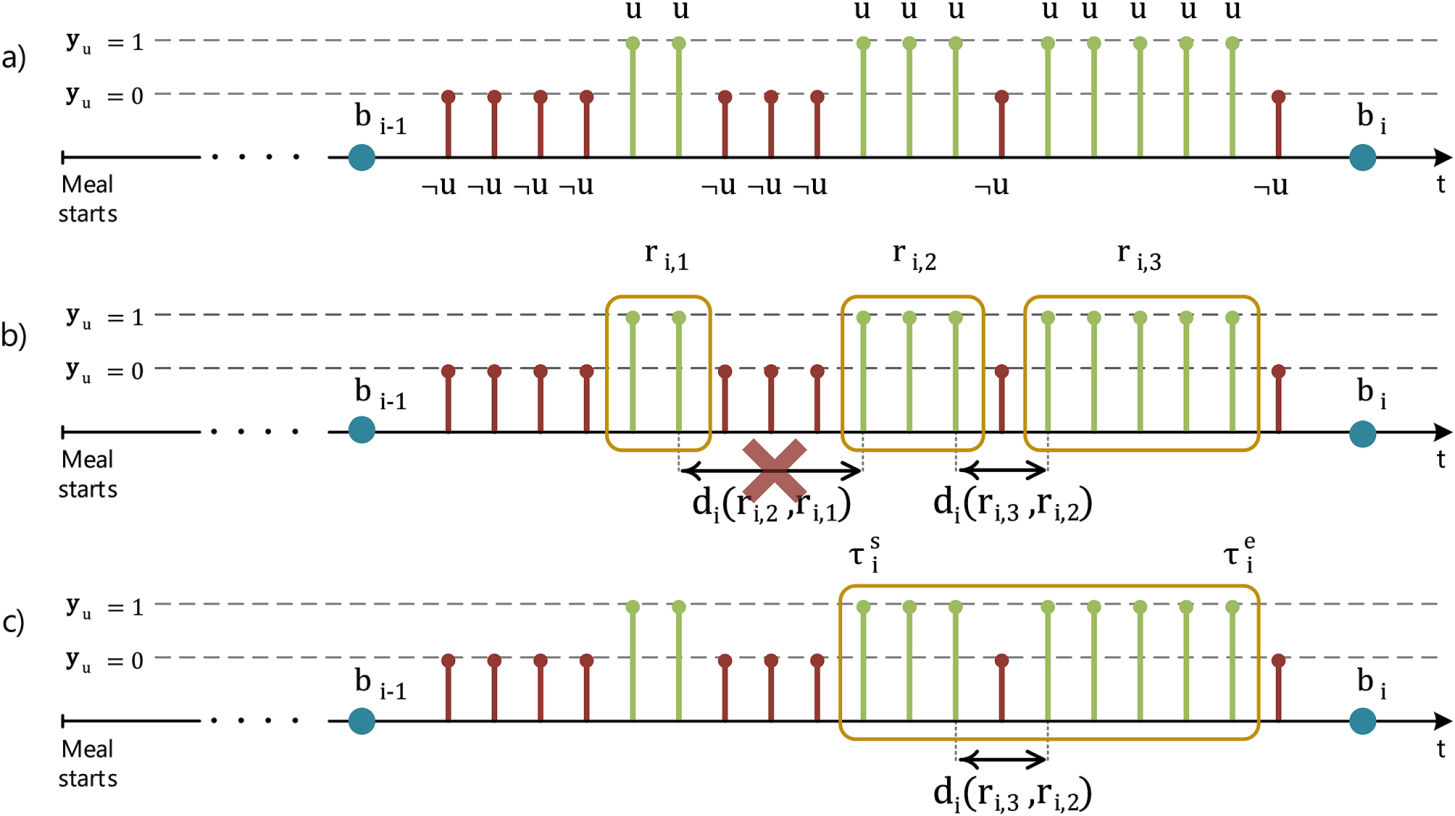
Example showcasing the steps towards the calculation of the PtM duration. In the first step (**a**) the subset $\mathbf{y}_{i,u}$ is presented for the inter-bite interval $[b_{i-1},b_{i}]$. Bite timestamps $b_{i-1}$ and $b_{i}$ are represented as blue circles. The green stems marked with *u* symbolize the upwards moments, while the red stems marked with $\lnot$*u* signify the non-upwards moments. In the following step (**b**) the set of u-regions $\mathfrak{R}_{i} = \{r_{i,1},r_{i,2},r_{i,3}\}$ is formed and the distances $d_{i}(r_{i,3},r_{i,2})$ and $d_{i}(r_{i,2},r_{i,1})$ are calculated. For this example we will use the $\times$ symbol to indicate that $d_{i}(r_{i,2},r_{i,1})$ is greater than the $\lambda _{d}$ threshold and therefore, that merge is considered unsuccessful. As a result, the number of consecutive successful merges $J_{i}$ equals to one. The last step (**c**) presents the temporal positioning of the $\tau _{i}^{s}$ and $\tau _{i}^{e}$ moments. Finally, duration $\text {PtM}_{i}$ is given by $\tau _{i}^{e} -\tau _{i}^{s} - d_{i}(r_{i,3},r_{i,2})$.

PtM extraction is repeated for all possible *U* inter-bite intervals, $[0,b_{1}], [b_{1},b_{2}],\ldots ,[b_{U-1},b_{U}]$, in a given session. It should be noted that PtM extraction for the inter-bite interval *i* may be discarded if the distance between $\tau _{i}^{e}$ and $b_{i}$ is greater than a threshold $\lambda _{b}$, or not completed at all if $\mathfrak{R}_{i}=\{\emptyset \}$. Experimentation with a small part of the EaC dataset allowed us to select 5 s as the value for $\lambda _{b}$; however, this parameter has minimal effect. At the end of the process the set of the PtM periods for the meal is created, formally defined as $\mathfrak{P}=\{\text {PtM}_{1},\text {PtM}_{2},\ldots ,\text {PtM}_{U'}\}$, with $U'\le U$. Figure 2 presents a visual example towards the calculation of the PtM duration for a given inter-bite interval.

### Subject-level Plate-to-Mouth representation

Given $\mathfrak{Q} = \{\mathbf{S}_{1},\mathbf{S}_{2},\ldots ,\mathbf{S}_{P}\}$, with $P \in \mathbb {Z}^{+}$ indicating the total number of meals originating from a *single* individual, subject-level representation is achieved in the following fashion. Initially, each of the $i=1,\ldots ,P$ sensor meal matrices $\mathbf{S}_{i}$ is transformed into PtM period set $\mathfrak{P}_{i}$, and the set $\mathfrak{Q}$ takes the following form: $\{\mathfrak{P}_{1},\mathfrak{P}_{2},\ldots ,\mathfrak{P}_{P}\}$. By *unfolding* each of the meal PtM period sets $\mathfrak{P}_{i}$ we obtain the final vector $\mathbf{x} = [\text {PtM}_{1,1},\text {PtM}_{1,2},\ldots ,\text {PtM}_{1,U_{1}'}, \ldots ,\text {PtM}_{P,1},\text {PtM}_{P,2},\ldots ,\text {PtM}_{P,U_{P}'}]$ with length $l_{\mathbf{x}}$ given by $\sum _{i=1}^{P}U_{i}'$. Each of the $\text {PtM}_{j,i}$ elements of $\mathbf{x}$ corresponds to the PtM duration of the *i*th bite belonging to the *j*th meal of the individual.

The subject-level representation is given by initially estimating the underlying Probability Density Function (PDF) of $\mathbf{x}$ by means of Kernel Density Estimation (KDE). The PDF is estimated using a Gaussian kernel and a bandwidth ${\hat{h}}$ calculated using Silverman’s rule of thumb^50^ presented below:

2 $$\begin{aligned} {\hat{h}}_{\mathbf{x}} = 0.9 \, \min \bigg ({\hat{\sigma }}_{\mathbf{x}}, \frac{{\hat{R}}_{\mathbf{x}}}{1.34} \bigg )\, l^{-1/5}_{\mathbf{x}} \end{aligned}$$

where ${\hat{\sigma }}_{\mathbf{x}}$ and ${\hat{R}}_{\mathbf{x}}$ are the variance and interquartile range (IQR) estimates of $\mathbf{x}$.

**Figure 3:**
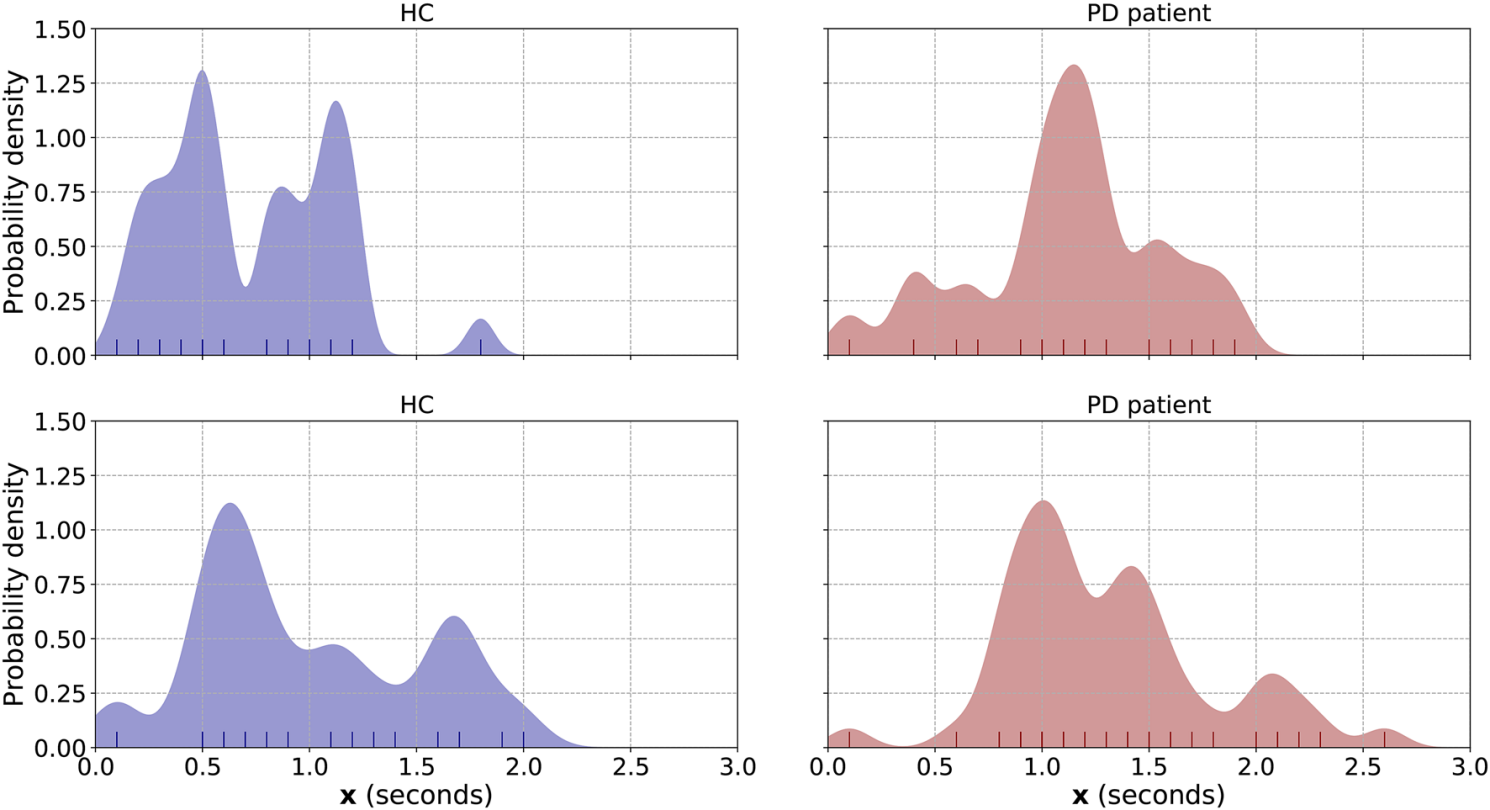
Figure depicting the subject level representations $\mathbf{x}_{f}$ for two HC (left column, blue) and two PD patients (right column, red). The vertical lines at the bottom of each figure denote the elements of $\mathbf{x}$ (all PtM durations from every meal of the participant) that where used to estimate the PDF in each case. Finally, the calculated bandwidths ${\hat{h}}_{\mathbf{x}}$ for the HC are 0.064 (top) and 0.120 (bottom), while for the PD patients are 0.092 (top) and 0.095 (bottom).

Finally, the subject-level representation $\mathbf{x}_{f}$ is given by evaluating the estimated PDF for a total of $l_{f} = 50$ points, equally-spaced in the interval between [0, 3] s. Figure 3 presents four indicative subject-level representation ($\mathbf{x}_{f}$) examples.

## Datasets

**Figure 4:**
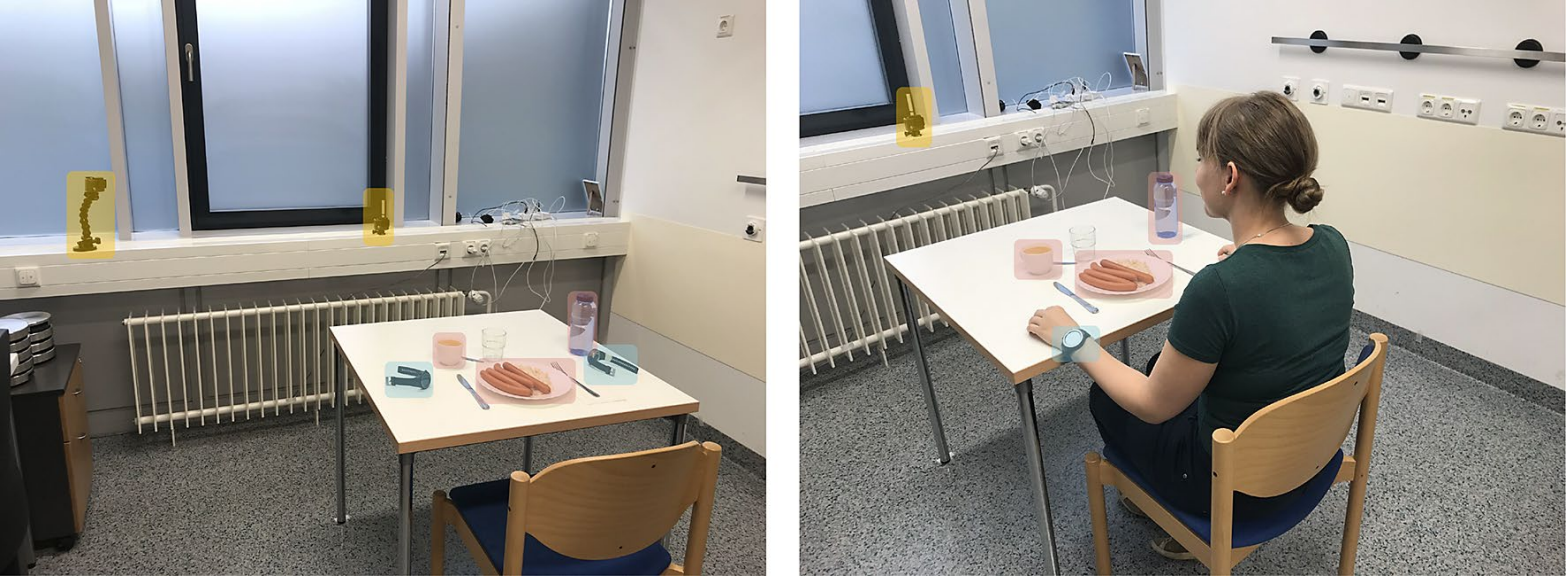
Figures depicting the EaC experiment setup. The picture on the left-hand side highlights the position of the two cameras (yellow). On the right picture, the researcher showcases the position of the smartwatch on the wrist (blue). In both pictures, the contents of the standardized meal are marked (red).

In the scope of this study we investigated the performance of the PtM indicator in three datasets, one collected in a controlled, in-the-clinic, environment and two collected *in-the-wild*. We will use the term in-the-wild to refer to uncontrolled settings that are outside of the clinic (e.g., private residences). All datasets were collected within the sphere of the i-PROGNOSIS Project (additional information is available online at: http://www.i-prognosis.eu), an H2020 EU-funded collective effort towards the early detection of Parkinson’s disease using commercial handheld and wearable devices. The EaC and EaH datasets were collected during the more exploratory EBePa study of the project, while SaH was collected during the main SData study. All subjects signed an informed consent prior to their participation. The researcher that appears in Fig. 4 signed an informed consent regarding the publication of her image in an online open-access journal. In addition, the recruitment and experimental procedures were performed according to the institutional and international guidelines on research involving adult human beings. The EaC and EaH data collections have been approved by the ethical committee of the Technical University Dresden (TUD) in Germany (EK75022018), while the SaH collection has been approved by the ethical committees of the King’s College Hospital in the UK (18/LO/0074) and TUD in Germany (EK451112017). All study participants have been investigated for baseline characteristics including general medical as well as PD specific history and medication intake. Furthermore in all study participants, PD patients and HC, an UPDRS assessment in medical ON condition has been performed by movement disorders specialists as well as PD specific validated scales and questionnaires were used to comply with the diagnostic criteria for PD^8^. Hereby the diagnosis of PD or healthy was provided to each study participant by movement disorders specialists as gold standard. On a more technical note, both the EaC and EaH IMU signals were recorded using the same software that was developed by our group while the main i-PROGNOSIS application was used to collect the IMU signals in SaH. Finally, the Huawei watch 2 was used to capture the 6 (Degrees of Freedom) DoF IMU signals at a rate of 100 Hz in all three datasets.

**Table 1:** Table presenting the technical and demographical information from the three datasets that were used in the scope of this work—namely, EaC, EaH and SaH.

Dataset	EaC		EaH		SaH	
Population	HC	PD	HC	PD	HC	PD
Subjects (N)	7	21	4	10	3	3
Age (a/s)	59.42/5.77	66.14/8.11	61.75/7.49	65.20/8.25	63.66/13.19	60.33/11.26
Gender (m/f)	2/5	12/9	1/3	6/4	1/2	1/2
Num. of meals (a/s/t)	1.00/0.00/7	1.00/0.00/21	2.00/1.22/8	2.90/1.22/29	107.00/128.69/321	102/67.89/308
Num. of bites (a/s/t)	33.14/7.31/232	39.52/15.64/830	31.25/7.86/257${\dagger }$	36.96/12.96/1072${\dagger }$	41.75/17.18/13,404${\dagger }$	57.63/18.75/17,752${\dagger }$
Meal duration sec (a/s/t)	427/80/2989	568/262/11,936	632/158/5059	732/338/21,247	1256/509/403,304	991/361/305,270
PD medication intake (y/n)	–	16/5	–	8/2	–	1/2
PD stage (early/advanced)	–	9/12	–	5/5	–	1/2

For each one of the datasets, information is provided regarding the PD and HC populations separately. It should be noted that in the case of in-the-wild datasets (EaH and SaH) no ground truth (GT) information exists in terms of number of bites. For the purpose of completion, we provide this information on calculations based on the number of *detected* bites (signified by the ${\dagger }$ symbol next to the cell). Meal duration is calculated as the difference of the timestamps between the last and the first bite (GT bites for EaC and detected bites for the in-the-wild datasets). Finally, the notation a/s/t is used to signify average/standard deviation/total, a/s for average/standard deviation, y/n for yes/no and m/f for male/female.

### In-the-clinic dataset

The clinical dataset, named EBePa-at-Clinic (EaC), was collected at the Department of Neurology of the Technical University Dresden (TUD) in Germany. The EaC dataset contains a total of 28 meal sessions from 28 subjects (i.e., one meal per subject). Out of the 28 subjects, 7 are HC and 21 are PD patients. For the data collection, participants were invited to the Department of Neurology of TUD to eat a provided standardized meal in a quiet room dedicated to the experiment around their usual lunch time (11:00–15:00) during a weekday. The meal included 200 g of pre-heated sausages, 400 g of cold potato salad, 200 g of apple puree and a bottle containing 500 ml of water to drink during the meal freely, a representative German meal.

Prior to commencing the meal, subjects were asked to wear two smartwatches, one on each wrist, and perform a single hand clapping motion in order to synchronize the video and inertial streams. Video capturing was achieved by using two Gopro Hero 5 cameras, one with a frontal and one with an angled viewpoint. No other specific instructions were given to the subjects, apart from notifying the supervising researchers when they have finished their meal. Participants were free to eat and drink as much as they wanted, at their own pace and without time constrains. Video recordings were analyzed independently in two different centers (AUTH and KI) to provide bite GT information (true timestamps of bite events) as well as the start and end points of upwards hand motions that lead to a bite. The EaC dataset sums to over 4 h of IMU recordings with a total of 1062 performed bite events (according to the video GT). Detailed technical information about the EaC dataset and the subject demographics can be found in Table 1, while Fig. 4 depicts the clinical experiment setup.

### In-the-wild datasets

In addition to the clinical data (EaC dataset in section "In-the-clinic dataset") we also collected two datasets from in-the-wild settings, namely EaH and SaH.

The EBePa-at-Home (EaH) dataset is the in-the-wild extension of the EaC dataset. More specifically, the majority of EaC participants were given a smartwatch to take home for seven days, and to wear it on the wrist of the hand that they use to operate the spoon/fork during their main warm meals. The intersection between EaH and EaC datasets (i.e., EaH $\cap$ EaC) is 12 participants (3 HC and 9 PD patients), which corresponds to a Jaccard Index (JI) of 0.4. Out of the 14 participants in EaH, 4 are HC and 10 are PD patients. In total, EaH contains 37 meal sessions summing up to a total duration of more than 7 h of IMU recordings.

As already mentioned, SData-at-Home (SaH) was collected as part of a different study within i-PROGNOSIS. As a result of this, no overlap exists between SaH and either the EaC or EaH. In more detail, SaH contains meal recordings from 6 participants, 3 HC and 3 PD patients. Despite of containing less subjects than the EaH or EaC counterparts, the SaH dataset incorporates significantly more meals, as the smartwatches were provided for approximately 3 months. In particular, SaH contains a total of 629 sessions. The total duration of IMU recordings in SaH is approximately 200 h.

In both in-the-wild datasets, the subjects were instructed to wear the smartwatch on the wrist of the hand that they use to operate the spoon/fork and enable data recording prior to the beginning of the main meal. Participants were instructed to disable the recording process after they finished eating. No other instructions were given and the participants were free to eat their meal of preference, at their own pace. Detailed technical information about the EaH and SaH datasets and their subject demographics can be found in Table 1.

## Evaluation

In addition to the EaC/EaH and SaH datasets, we make use of the, previously collected and analyzed, external *Food Intake Cycle* (FIC) dataset in order to train the SVM array and the RNN that are responsible for the recognition of micromovements and the modeling of their temporal evolution, respectively. The FIC dataset contains the 3D acceleration and orientation velocity signals from 21 meal sessions from 12 HC. Meals were recorded under realistic conditions in the cafeteria of the Aristotle University of Thessaloniki using commercial smartwatches. More specifically, the Microsoft band 2 was used to record ten out of the twenty-one meals (at a rate of 62 Hz) and the Sony smartwatch 2 for the remaining meals (at a rate of 200 Hz). Based on the supporting videos, FIC also includes detailed annotations regarding wrist gestures (e.g., wrist moving upwards) and the exact bite moments. The total number of annotated food intake cycles (i.e., bite events) in FIC sums to 1332. It should be emphasized that there is no overlap between the subjects of the FIC and the EaC, EaH and SaH datasets. In depth information about the FIC dataset can be found in a previous work of our group^47^.

The extraction of a meal’s PtM durations depends heavily on the first part of the proposed algorithm, i.e., the upwards micromovement and bite moments detection. This is because the meal’s PtM durations are calculated independently for all inter-bite intervals. As a result, for the first experiment (**EXI**) we evaluate the bite detection effectiveness in a train/test split fashion. More precisely, we use the EaC dataset as the test set and the FIC dataset as the train set.

Goal of the next experiment (**EXII**), performed in the EaC dataset, is to evaluate the quality of the smartwatch-extracted PtM durations. More specifically, in EXII we compare the PtM durations as extracted by the proposed method using the smartwatch 6 DoF IMU data, against the PtM durations that are extracted by manual ground truth annotation produced by two independent researchers analyzing the meal videos.

In the third series of experiments (**EXIII**) we evaluate the performance of the proposed method and specifically, how well the extracted subject-level PtM indicator can discriminate between the eating behavior of PD and HC. The diagnosis of PD or HC was provided by movement disorders specialists during clinical evaluations based on a standardized protocol. In every experiment under EXIII we follow a Leave-One-Subject-Out (LOSO) cross-validation scheme, meaning that in each repetition of the experiment we use the data of all but one subject as the training set and the left-out subject as the test set. The experiment ends when all subjects in the dataset have been left-out once. In more detail, we performed four LOSO experiments to evaluate the performance of the proposed subject-level indicator. Experiments EXIII-A and EXIII-B make use of the EaC and EaH datasets, respectively. For EXIII-C we use the dataset that result from the intersection of subjects between EaC and EaH (i.e., EaC $\cap$ EaH). Finally, for EXIII-D we employ the SaH dataset.

## Results

For all experiments, we selected $\lambda _{d}$ to be equal to 0.5 s. We support this choice as it approximates the median duration (0.483 s) of non-upwards movements that occur between upwards movements that contribute to the bite’s PtM, according to the video GT of the EaC dataset. In addition, experimentation with a small part of the EaC dataset allowed us to select 5 s as the value for $\lambda _{b}$; however, this parameter has minimal effect. Finally, in order to deal with the incompatibilities in the sensor sampling frequency among the different datasets (EaC, EaH, SaH and FIC), all data were resampled to a constant rate of $f_{s}=100$ Hz.

**Figure 5:**
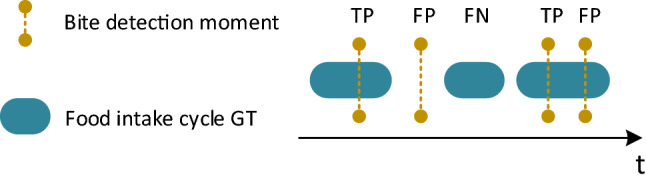
Representative examples of the evaluation scheme^47^ that was used to produce the results in EXI. The first bite within a GT interval counts as a True Positive (TP), any further detected bites count as False Positives (FP). In addition, detected bites outside GT intervals also count as FP. Empty GT intervals without any detected bites count as False Negatives (FN). It should be noted that the adopted evaluation method cannot calculate the number of True Negatives (TN).

Table 2 presents results of EXI in the form of a confusion matrix. Essentially, the confusion matrix reflects the performance of the micromovement and bite detection part of the algorithm when trained with a dataset that solely contains HC (the FIC dataset) and evaluated using a dataset that contains a combination of HC and PD patients. Performance metrics are produced by adopting the strict evaluation scheme^47^ (representative examples are depicted in Fig. 5) which allows for a single detected bite to be considered as true positive in a GT bite interval. By using the number of True Positives (TP), False Positives (FP) and False Negatives (FN) we can calculate the precision, recall and F1 metrics which are equal to 0.929, 0.975 and 0.952, respectively. It should be noted that it is critical to obtain satisfactory results in EXI as the estimation of the PtM indicator (and as a result the outcome of all upcoming experiments) depends on the quality of the micromovement and bite moment detection.

**Table 2:**
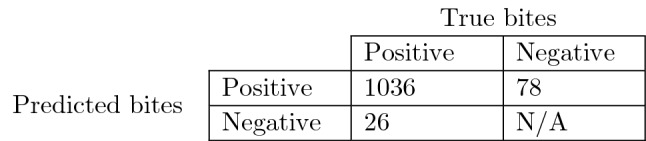
Confusion matrix depicting the bite detection performance of EXI using the EaC dataset.

N/A (i.e., Not Available) is used to indicate TN cannot be calculated using the adopted evaluation scheme^47^; this is depicted in Fig. 5. The total number of true bites in EaC is 1062 (sum of TP and FN). Given the confusion matrix, the performance metrics precision/recall/F1 are calculated to be 0.929/0.975/0.952.

**Figure 6:**
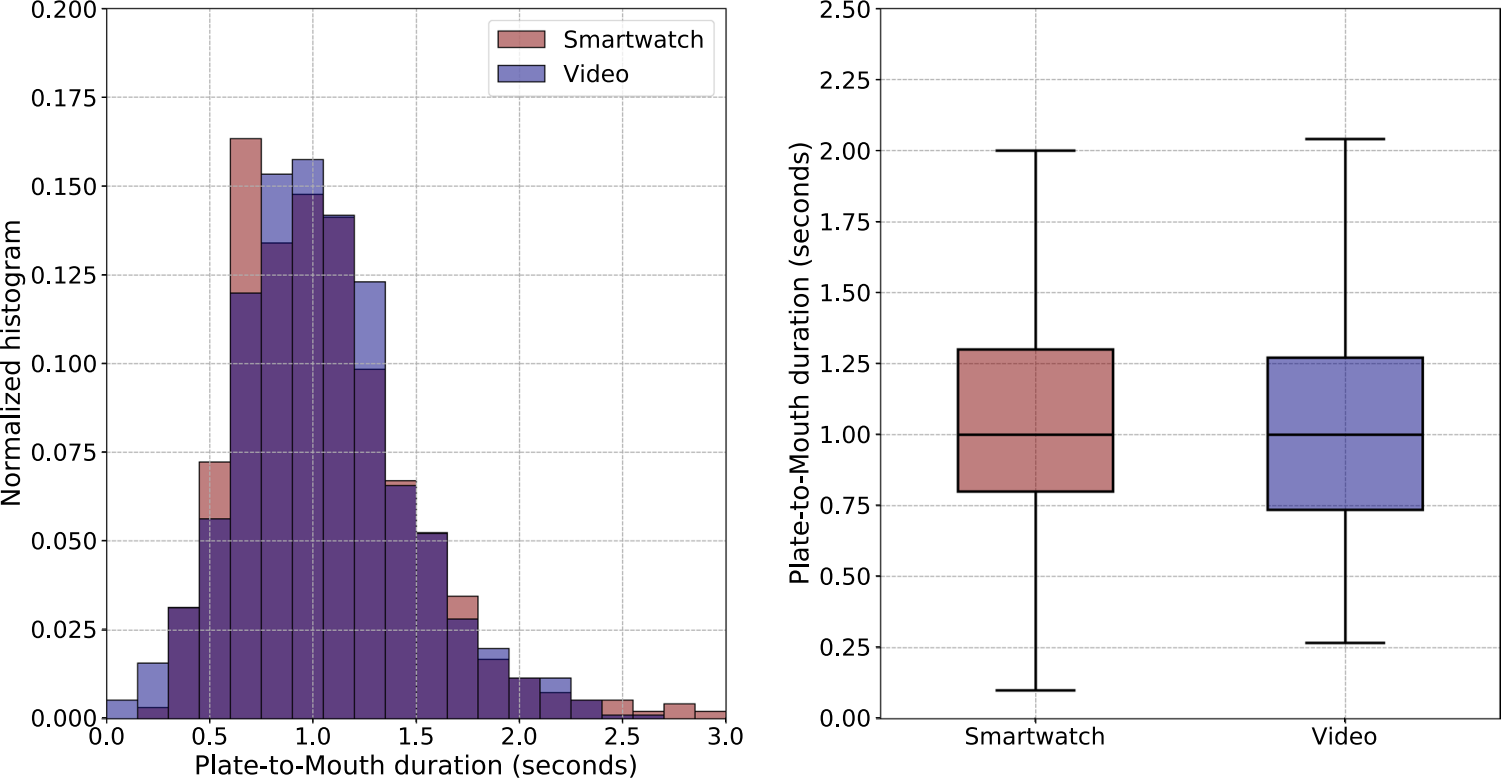
Comparison of the video and smartwatch-based extraction of Plate-to-Mouth durations ($n=1036$) in the context of EXII (EaC dataset). The normalized superimposed histograms (left) and box plots (right) showcase the large overlap between the two distributions. Regarding the left figure, the dark blue color represents the overlap between the two histograms.

Given the fact that bite detection performance is not flawless (this is reflected in Table 2), the direct, one-versus-one, comparison of the smartwatch-based and video-based PtM durations is not feasible. For this reason, we performed EXII using the PtM durations for the $n = 1036$ bites (i.e., the number of TP from Table 2) that are correctly classified. As a result, the Mean Squared Error (MSE) and the Mean Average Error (MAE) where found to be 0.214 and 0.310 s, respectively. Figure 6 depicts the video and smartwatch-based PtM duration distributions. The Pearson’s correlation coefficient for the GT annotations between the two independent raters, indicating the inter-rater agreement, was $r=0.902$ (with $\rho <0.001$) for average meal-level PtM durations and $r=0.755$ (with $\rho <0.001$) for one-versus-one bite-level PtM durations.

**Figure 7:**
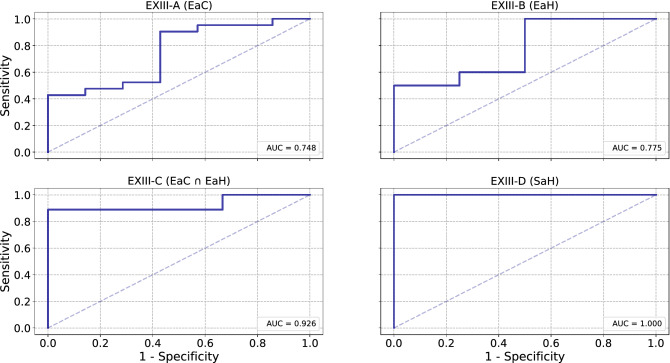
Figure presenting the ROC curves (1-specificity against sensitivity) for the four experiments under EXIII. The obtained AUC metrics are also provided in the bottom right corner of each sub-figure. The presented ROC curves illustrate how well the extracted subject-level PtM indicator can be used to discriminate between the eating behavior of PD patients and HC.

Having obtained satisfactory results from EXI and EXII, we proceed with the EXIII series of experiments. In particular, experiments EXIII-A, EXIII-B and EXIII-D deal with the classification of eating behavior profiles to the PD or the HC populations given the EaC/EaH/SaH datasets. We report an Area Under the Curve (AUC) of 0.748, 0.775 and 1.000 for EXIII-A, EXIII-B and EXIII-D, respectively. In EXIII-C we perform classification by only taking into consideration the meals from the subjects that participated both in EaC and EaH (EaC $\cap$ EaH). For EXIII-C we report an AUC of 0.926, which hints that a subject’s PtM-based eating behavior profile can be more accurate if the subject has contributed more meals. Figure 7 depicts the Receiver Operating Characteristic (ROC) curves, while Table 3 aggregates the obtained results and presents them in two different operating points, one requiring high sensitivity ($\ge 0.85$) and one requiring high specificity ($\ge 0.85$). In all experiments under EXIII, classification is achieved by using a binary SVM with the RBF kernel and regularization parameter *C* equal to 1. The subject-level indicators that belong to PD patients constitute the positive class and the ones that belong to HC the negative class. Furthermore, in order to resolve the imbalance between the two classes (HC and PD) during training, for all experiments each class was proportionally weighted based on it’s prior probability.

**Table 3:** Table aggregating the results from the series of experiments under EXIII; essentially, how well the subject-level PtM indicator can be used to discriminate between the PD and HC populations.

Operating Point	Experiment	Specificity	Sensitivity (recall)	Precision	F1
Sensitivity $\ge 0.85$	EXIII-A	0.571	0.904	0.863	0.883
	EXIII-B	0.500	1.000	0.833	0.909
	EXIII-C	1.000	0.888	1.000	0.941
	EXIII-D	1.000	1.000	1.000	1.000
Specificity $\ge 0.85$	EXIII-A	0.857	0.476	0.909	0.625
	EXIII-B	1.000	0.500	1.000	0.667
	EXIII-C	1.000	0.888	1.000	0.941
	EXIII-D	1.000	1.000	1.000	1.000

The obtained results are presented in two operating points, one requiring high sensitivity (upper half) and one requiring high specificity (lower half).

## Discussion

**Figure 8:**
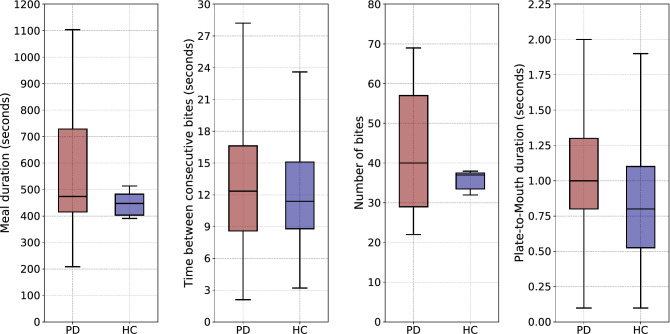
Box plots depicting (from left to right) how: (**i**) the duration of meals, (**ii**) the time between consecutive bites, (**iii**) the number of bites and (**iv**) the PtM durations extracted from all inter-bite intervals, are distributed to the PD and HC populations of the EaC dataset.

There is a single UPDRS item which *subjectively* asks PD patients to evaluate their ability to use eating utensils. This UPDRS part II item 9 covers different aspects of food handling such as speed, the ability to cut food, fine motor skills and the need for assistance. As a consequence, it is a rough item which does not cover the different aspects of food intake in detail neither separately. According to a recent study^51^, indices that are commonly used to model and determine eating behavior are: (1) the meal duration, (2) the time between bites (i.e., eating rate), and (3) the number of bites. Even though the meal duration, the eating rate and the number of bites can be *objectively* measured, early experimentation using the EaC dataset revealed that those three metrics do not allow for separation between the PD and HC populations. This is illustrated in Fig. 8. The figure showcases the large overlap between the PD and HC populations regarding the meal duration, the time between consecutive bites and the number of bites. It should be mentioned that despite the large overlap between the two populations in the cases of meal duration and number of bites, the range of values for the HC population is much more narrow than the PD one. In addition, the rightmost part of Fig. 8 demonstrates how the PtM durations extracted from all inter-bite intervals differ between the two populations. More specifically, it can be seen that approximately $50\%$ of the PtM durations that belong to the HC distribution are outside the Interquartile Range (IQR) of the PD distribution. The methodology presented in this paper allows for the objective measurement of PtM, in clinical and in-the-wild settings.

The obtained results presented in the "Results" section showcase the high performance of the proposed methodology towards the classification of eating behavior profiles to the PD and HC populations. More specifically, EXI results show that the food intake detection algorithm generalizes well on the EaC dataset (the only dataset among EaC, EaH and SaH with video GT) when trained using the external, and non-overlapping, FIC dataset (F1 score of 0.952). Since PtM is calculated for all inter-bite intervals within a meal, high performance of the food intake detection part of the methodology is crucial in order to obtain reliable PtM estimates. Results from EXII demonstrate that smartwatch-based PtM extraction approximates the video-based one, with an MSE/MAE of 0.214/0.310. Having obtained satisfactory results from EXI and EXII, we proceed with the EXIII series of experiments. In particular, experiments EXIII-A/-B/-D deal with the classification of eating behavior profiles to the PD or the HC populations given the EaC/EaH/SaH. We report an AUC of 0.748, 0.775 and 1.000 for EXIII-A, EXIII-B and EXIII-D, respectively. In EXIII-C we perform classification by only taking into consideration the meals from the subjects that participated both in EaC and EaH (EaC $\cap$ EaH). For EXIII-C we report an AUC of 0.926, which hints that a subject’s PtM-based eating behavior profile can be more accurate if the subject has contributed more meals.

In our previous work^26^ we introduced a less elaborate adaptation of the PtM indicator and performed experiments using solely the EaC dataset. However, even the simpler version of the proposed indicator uncovered the high potential of the initial idea. Early experimental results revealed an F1 score of 0.789 (compared to the 0.883 of the current approach presented in Table 3, high sensitivity operating point of EXIII-A) towards the correct classification of eating behavior profiles to the PD or healthy population, using a simple one-dimensional optimal threshold scheme (according to Otsu’s method^52^).

Both our previous^26^ and current work attempt to objectify the in-meal eating behavior of PD patients and HC by extracting indicators using IMU sensor data originating from commercial smartwatches. The reasons behind selecting the smartwatch as the sensor platform of choice is that modern wearable technologies, such as smartwatches, are widely available and increasingly used by the general population. In addition, they require minimal effort to wear and handle and do not raise suspicion as to being a medical monitoring device, thus reducing social stigma^53^. Distributed via *application stores*, an application could be made publicly available that screens for alterations in eating behavior. This offers an opportunity to screen a large group of healthy individuals who may manifest symptoms compatible with a diagnosis of prodromal PD. In addition to screening the general population, alterations in eating behavior can be monitored, via remote sensing, in those with a PD diagnosis using a continuous assessment approach during treatment. The methodology described in this study is capable of monitoring the temporal evolution of eating behavior profiles, a largely neglected aspect of PD symptomatology, and allow clinicians to keep track of the progression of this motor issue and indirectly monitor efficacy of dopaminergic therapy as well as side effects.

A limitation of our approach is that in-the-wild meal recordings require input from the participants. Specifically, the participant needs to initiate and terminate data recording prior and after each meal. Such manual collection configuration can lead to sparsity in the collected dataset. However, a past work of our group^49^ exploits all-day IMU recordings and introduces a bottom-up method that uses the distribution of bites during the day to effectively detect meal start and end moments. An additional limitation of the current method is that it can exhibit unpredictable behavior when the subject performs drinking gestures or eats without the fork and/or the spoon. This is attributed to not have introduced instances of eating with e.g., bare hands or chopsticks to the SVM and LSTM mechanisms during the training process. Obtaining a dataset that is more diverse in the use of eating utensils and contains drinking gestures will allow to resolve this unpredictable behavior. Finally, it should be noted that the quality of the extracted PtM durations depends heavily on the bite detection performance. This is because PtM is calculated for all periods in-between bites during the course of a meal. As it can be seen from Table 2, bite detection performance is high (precision/recall/F1 equal to 0.929/0.975/0.952) but not without errors; this can cause erroneous measurements in situations where the subject performs limited amount of bites and/or meals.

In addition, an incident that was observed in the EaC dataset using the video GT, is that in rare occasions PD participants were leaning their upper body towards the surface of the table. Essentially, this means that a simple, small rotation of the wrist is enough to transfer food from the plate to the mouth (no acceleration is registered by the sensor). Such food intake gestures can go unnoticed from the upwards micromovement recognition (and bite detection) mechanism and have an immediate effect on the extraction of the PtM indicator. However, despite the appearance of such sitting postures, the obtained bite detection performance in EXI (which is directly affected by the micromovement recognition quality) and the comparison of video- and IMU-based PtM durations in EXII (which is affected by both the micromovement recognition and the bite detection quality), is proven to be satisfactory (F1 of 0.952 for EXI and MSE/MAE of 0.214/0.310 for EXII). The way different sitting/eating postures can affect the extracted indicators is a complex topic and we consider it as a future direction for the presented work.

## Conclusions

In this paper we have defined PtM, an indicator that relates with the time spent for the hand that operates the utensil to transfer a quantity of food from the plate into the mouth. Furthermore, we also presented a methodology towards the objective calculation of individual PtM durations and the creation of the subject’s in-meal eating behavior profile. Experimental results using three datasets (one in clinical and two in-the-wild settings) reveal the high potential of our approach towards the classification of in-meal eating profiles to the PD or the healthy populations. To this day, this is the first endeavor towards the introduction of an automatically estimated, eating behavior indicator for PD based on objective sensor measurements using commercial smartwatches.
